# *LMNA* Mutation in a Family with a Strong History of Sudden Cardiac Death

**DOI:** 10.3390/genes13020169

**Published:** 2022-01-19

**Authors:** Laura Keil, Filip Berisha, Dorit Knappe, Christian Kubisch, Moneef Shoukier, Paulus Kirchhof, Larissa Fabritz, Yorck Hellenbroich, Rixa Woitschach, Christina Magnussen

**Affiliations:** 1 Department of Cardiology, University Heart and Vascular Center Hamburg, 20251 Hamburg, Germany; f.berisha@uke.de (F.B.); d.knappe@uke.de (D.K.); p.kirchhof@uke.de (P.K.); larissa.fabritz@uke.de (L.F.); c.magnussen@uke.de (C.M.); 2 German Center for Cardiovascular Research (DZHK), Partner Site Hamburg/Kiel/Luebeck, 20251 Hamburg, Germany; 3 Institute of Human Genetics, University Hospital Hamburg Eppendorf, 20246 Hamburg, Germany; c.kubisch@uke.de (C.K.); r.woitschach@uke.de (R.W.); 4 Prenatal Medicine Munich, Department of Molecular Genetics, 80639 Munich, Germany; shoukier@praenatal-medizin.de; 5 Institute of Human Genetics, University of Luebeck, 23538 Luebeck, Germany; yorck.hellenbroich@uksh.de

**Keywords:** LMNA, dilated cardiomyopathy, heart failure

## Abstract

We report a family with heterozygous deletion of exons 3–6 of the *LMNA* gene. The main presentation of affected family members was characterized by ventricular and supraventricular arrhythmias, atrioventricular (AV) block and sudden cardiac death (SCD) but also by severe dilative cardiomyopathy (DCM). We report on two siblings, a 36-year-old female and her 40-year-old brother, who suffer from heart failure with mildly reduced ejection fraction, AV conduction delays and premature ventricular complexes. Their 65-year-old mother underwent heart transplantation at the age of 55 due to advanced heart failure. Originally, the *LMNA* mutation was detected in one of the uncles. This index patient and three of his brothers died of SCD as well as their father and aunt. The two siblings were treated with implanted defibrillators in our specialized tertiary heart failure center. This case report places this specific genetic variant in the context of LMNA-associated familial DCM.

## 1. Introduction

Mutations in the lamin A/C gene (*LMNA*) can be detected in one in twenty to one in twelve patients with dilated cardiomyopathy [1,2]. Patients with LMNA mutations typically present with arrhythmias and conduction disorders, including sudden cardiac death, prior to developing severe heart failure at an early age [1,3,4,5]. Importantly, for the clinical work-up, patients with LMNA mutations often present with early-onset arrhythmias or conduction disturbances prior to developing heart failure [4,6]. The sudden cardiac death (SCD) risk is already high in early stages [1,4]. Apart from DCM, *LMNA* mutations have also been associated with skeletal muscle disorders, such as Emery–Dreifuss or autosomal-dominant limb-girdle muscular dystrophy and progeroid phenotypes [1,7,8,9].

The protein encoded by *LMNA* is essential for stable construction and functioning of the nucleus [10]. It plays a major role in the regulation of gene expression in cardiomyocytes [11]. As a consequence, the expression of genes involved in cardiac electrical conduction may also be affected [12,13]. 

In this case report, we precisely phenotype three members of a family with an untypical *LMNA* mutation and relate this genetic pathology to different clinical manifestations.

## 2. Case Presentation

### 2.1. Clinical Presentation

**Figure 1:**
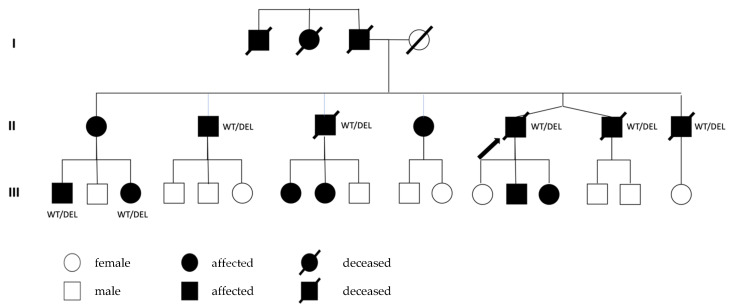
Pedigree of the family. The arrow marks the index patient. WT/DEL = wild type/deletion, confirmed mutation.

In 2010, a 19-year old patient (Figure 1, patient III:3) first presented to our department for treatment of atrioventricular (AV) nodal re-entrant tachycardia; catheter-based slow-pathway modulation of the AV node was performed. Postprocedural AV block I° was documented. In 2010, the patient suffered from paroxysmal atrial tachycardias with a duration of 10 min to 1 ½ h with sudden onset and accompanied from dizziness, palpitations and dyspnea. In electrophysiological testing, atrial tachycardia could be induced, but ablation was not possible due to short duration and self-limitation. Apart from supraventricular arrhythmia or conduction disturbances, no structural heart disease was present at that time point. Four years later, the patient presented various times with palpitations and was treated with flecainide as a “pill in the pocket”. Tele-electrocardiography documented the occurrence of supraventricular couplets. Echocardiography showed normal dimensions and function of both ventricles until the age of 29, while creatine kinase (CK) levels were slightly elevated at 362 U/L. 

**Figure 2:**
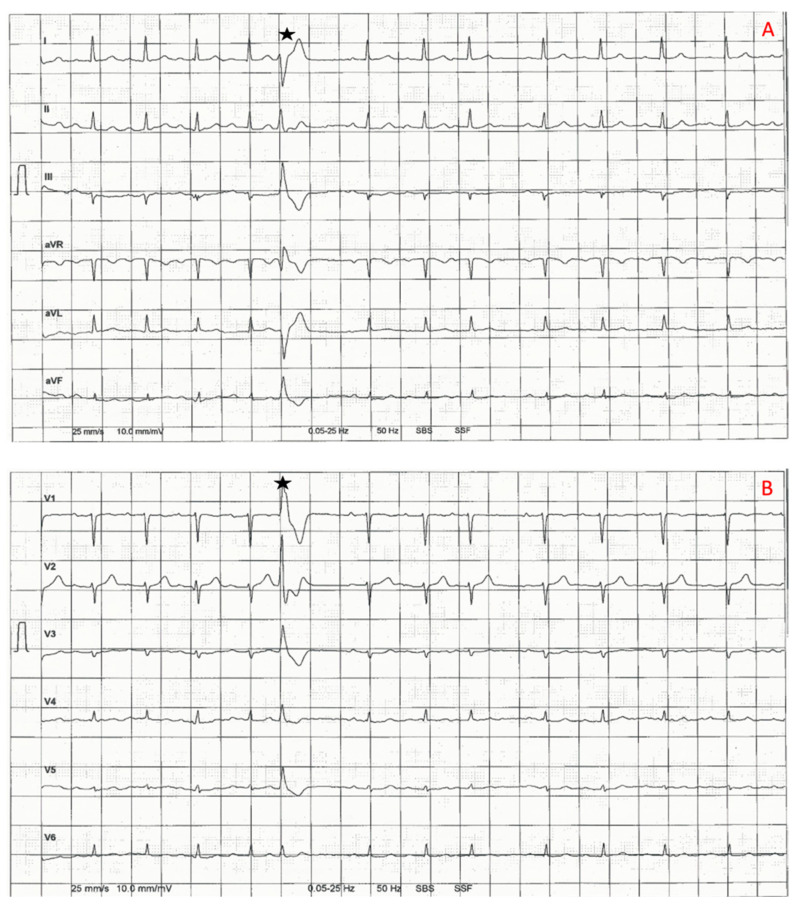

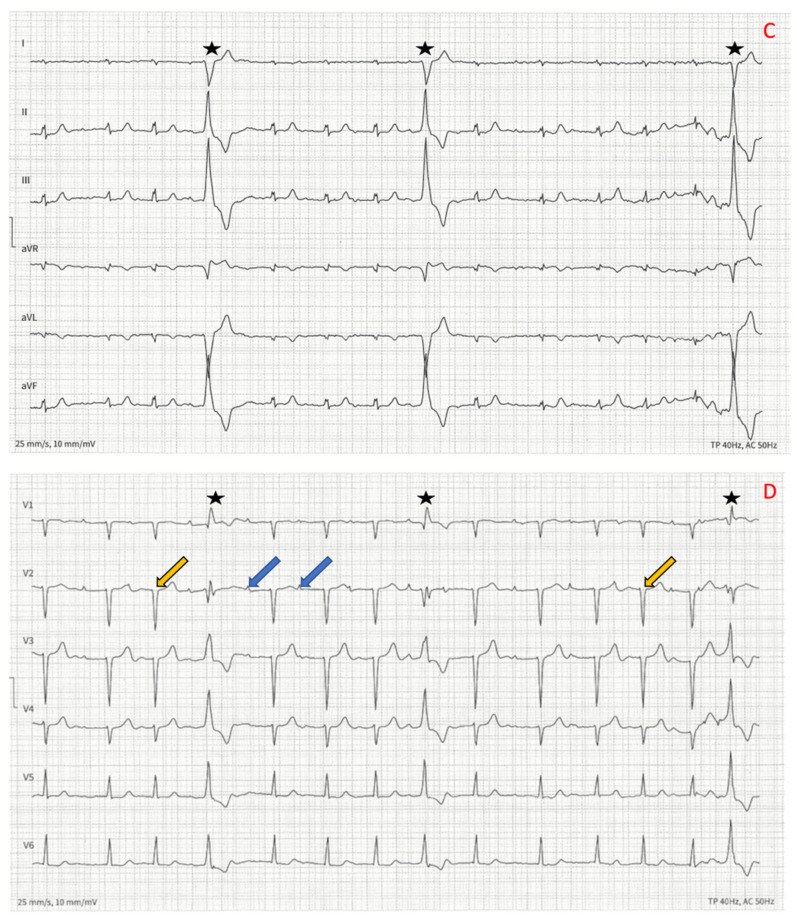
Electrocardiographic samples of patient III:3. (**A**) and (**B**) ECG from 2014, (**C**) and (**D**) ECG from 2021. Stars indicate premature ventricular complexes (PVCs). Blue arrows mark the p-waves, whereas orange arrows mark supraventricular premature beats. Duration of atrioventricular (AV) block and PVCs increase over time.

**Figure 3:**
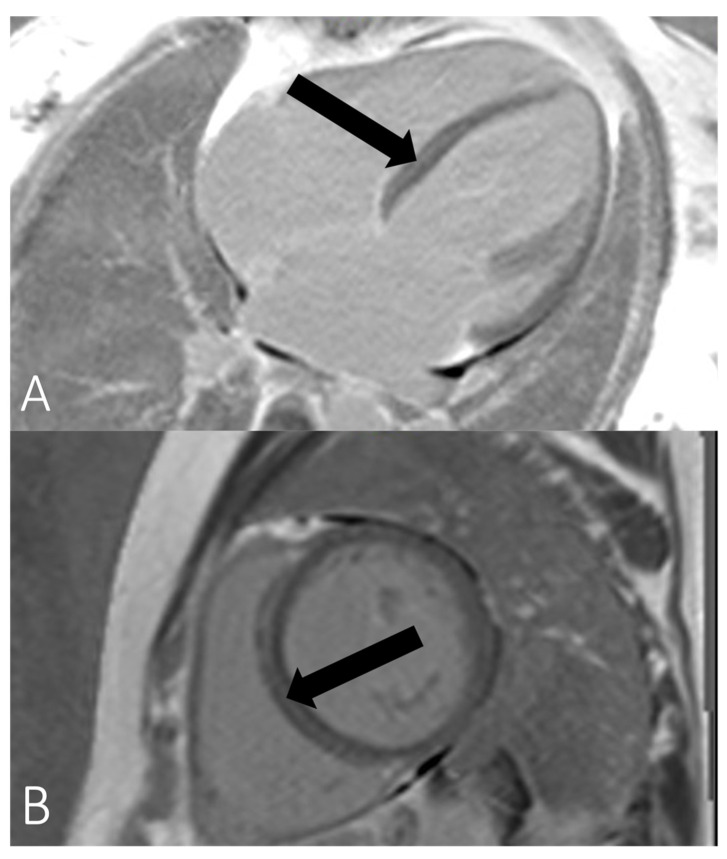
Magnetic resonance imaging (MRI) samples of patient III:3. Septal midwall late-gadolinium enhancement (LGE) in (**A**) long-axis view and (**B**) short axis view, indicated by black arrows.

The familial nature of heart disease was only identified when the patient presented again in 2021. The patient described shortness of breath and palpitations. In the electrocardiogram (ECG), premature ventricular complexes (PVC) were detected, and the previously known AV block I° had progressed from a PR interval of 0.24 s to 0.34 s (Figure 2). Holter-monitoring documented a PVC burden of 6.12% with a polymorphic pattern and one ventricular salve. Left ventricular function was now mildly reduced (EF 45–50%, see supplementary data Video S1A–C) while cardiac dimensions including left ventricular end-diastolic diameter (LVEDD) were normal, and N-terminal pro-B-type natriuretic peptide (NT-proBNP) level was elevated at 2358 pg/mL. Cardiac magnetic resonance imaging (MRI) was performed for further evaluation and showed septal, mid-myocardial late-gadolinium enhancement as well as elevated septal T1 time (see Figure 3). Genetic testing identified a heterozygous deletion in exons 3–6 of the LMNA gene (NM_170707.2). Testing was performed by next generation sequencing (NGS), and results have been futher confirmed by multiplex ligation dependent probe amplification (MLPA). The first rsk score for sudden cardiac death (SCD) was estimated at 35% in 5 years using the LMNA-risk VTA calculator [14], and a primary prevention defibrillator was implanted. Moreover, we deliberately chose cardiac resynchronization therapy (CRT), as we expected high right ventricular (RV) pacing due to higher AV block.

**Figure 4:**
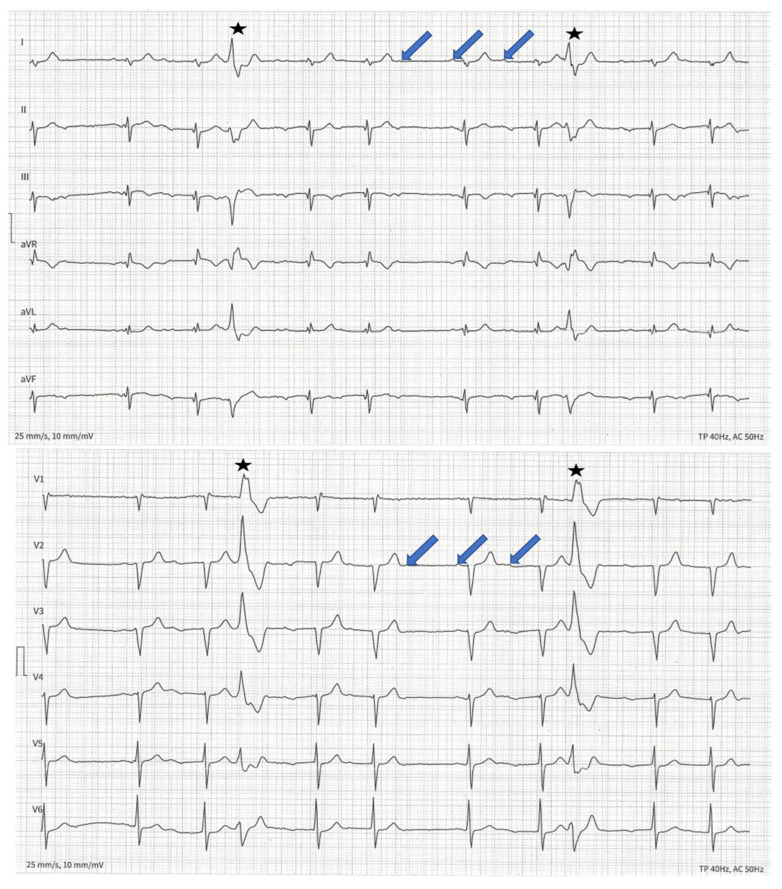
ECG of patient III:1. Arrows indicate p-waves; the PQ interval is increasing as with a second-degree type I AV block. Stars indicate PVCs.

Genetic testing of first-degree relatives identified the same mutation in her brother (Figure 1, patient III:1). He suffered from paroxysmal atrial fibrillation (AF) and dyspnea during exercise. Similar to his sister, left ventricular ejection fraction (LVEF) was 45–50%, LVEDD was normal and NT-proBNP and CK were elevated at 824 pg/mL and 337 U/L, respectively. Second-degree type I AV block and PVC burden of 5.8% were detected in ECG (Figure 4) and Holter-monitoring. MRI findings resembled his sister’s. Primary CRT-D was implanted due to a SCD risk score of 26% and expected high RV stimulation.

**Figure 5:**
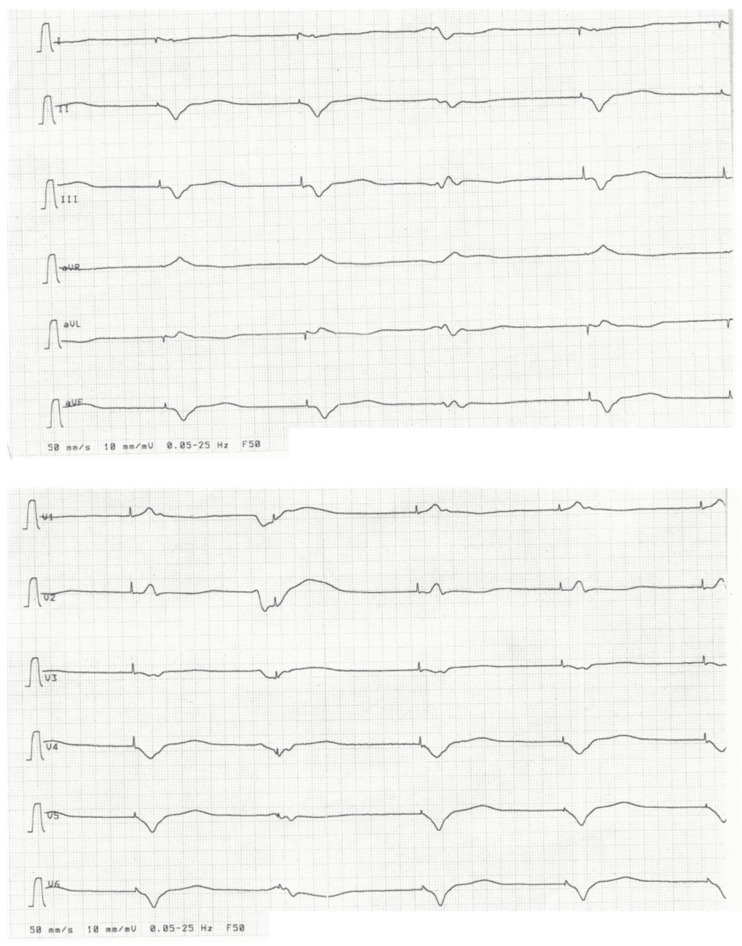
ECG of patient II:1 reveals continuous pacer stimulation; no p-waves are discernible as present bradyarrhythmia.

The mother of the two siblings, a 56-year-old female (Figure 1, patient II:1), presented at our department for initial assessments in 2011. She had signs of cardiac congestion (peripheral oedema, pleural effusion) and reported fatigue, dyspnea at a walking distance of 100 m (New York Heart Association (NYHA) III) and a general weakness. The electrocardiogram (ECG) documented pacer stimulation (see also Figure 5), echocardiography showed an ejection fraction (EF) of 35%, dilation of the left and right ventricle and severe tricuspid regurgitation. Spiroergometry revealed a maximal oxygen intake of 9.62 mL/min/kg and had to be stopped at 27 watts because the patient reached the anaerobic threshold. The patient’s history included implantation of a pacemaker in 2003 because of bradyarrhythmia and an upgrade to a defibrillator with cardiac resynchronization therapy (CRT-D) five years later due to heart failure with reduced ejection fraction (HFrEF, LVEF 18%). In 2011, directly prior to referral to our center, ventricular tachycardias led to adequate defibrillator shocks, and amiodarone therapy was started. Due to end-stage heart failure, the patient was listed for heart transplantation, which was successfully performed in 2012. Today, the patient is clinically stable, and echocardiography shows a good biventricular function.

### 2.2. Genetic Findings

The heterozygous deletion in exons 3–6 of the *LMNA* gene was first detected in an elder brother of patient II:1 (see Figure 1, arrow) by using a multi-panel gene analysis of 60 cardiomyopathy-genes and confirmed by MLPA. He died at the age of 56, shortly before his planned heart transplantation after a long history of heart failure. Three of his brothers died of SCD aged 44, 47 and 56. The latter was also diagnosed with DCM. Their father died at the age of 38 during exercise, and their aunt died at the age of 46.

Patient II:1 has not been genetically tested, as she has to be a mutation carrier. A living sister of patient II:1 is treated with an ICD but has not been genetically tested yet. An overview of the genotype for the known *LMNA* deletion in the family is given in Figure 1.

## 3. Discussion

In this case report, we described the clinical presentation of different members of a family with heterozygous deletion of *LMNA* exons 3–6. Besides a strong family history of SCD, the mother presented with end-stage heart failure due to DCM and a history of ventricular tachycardias (VTs). Her children exhibited AV conduction disturbances, a high rate of PVC, supraventricular tachycardias and only mildly impaired left ventricular function. Previous case studies reported a similar incidence of SCD, AF and AV blocks [15,16,17].

Due to *LMNA*’s critical role in nuclear stability, *LMNA* mutations were hypothesized to lead to reduced mechanical stress resilience in cardiomyocytes causing apotosis [18,19]. Subsequently, tissue replacement by fibrosis may act as an arrhythmic substrate [20]. Underlying molecular mechanisms have been mostly investigated in mouse models with *LMNA* missense mutations, and various pathological pathways have been described [19,21]. Chatzifrangkeskou et al. showed that transforming growth factor β-mediated activation of extracellular signal-regulated kinase ½ induces expression of the connective tissue growth factor, resulting into myocardial fibrosis [22]. Different mutations may be ensued by distinct molecular pathways requiring taylored therapies [19]. Nevertheless, the exact molecular mechanism leading to DCM and electrical disturbances is still poorly understood [6,11].

Deletion of whole exons of *LMNA* are extremely rare, and most variants are missense mutations [23]. The Human Gene Mutation Database lists seven mutations with gross deletions in total, of which four are whole exon deletions (Human Gene Mutation Database. Available online: http://www.hgmd.cf.ac.uk/ac/all.php, accessed on 13 December 2021). This particular mutation in our case led to a deletion of four exons.

Large deletions are assumed to generate a loss of function [24]. Gupta et al. described the case of a patient with deletion of exons 3–12 [25]. This patient exhibited non-sustained VTs and mild heart failure. Ultrastructural analysis of his endomyocardial samples showed a damaged nuclear envelope, and immunostaining revealed reduced protein expression [25]. Consequently, the authors suggested haplo-insufficiency and resulting impaired nuclear integrity as a possible underlying pathological mechanism for the development of DCM [25]. This may be transferred to our case with the deletion of exons 3–6 and hence probably partly reduced protein expression. Other studies found patients with deletion of exon 1, a deletion including the start-codon and a case with deletion of the whole LMNA gene [24,26,27]. The cardiac phenotype included DCM, AV block, VTs and ventricular fibrillation and SCD [24,26,27]. To our knowledge, deletion of exons 3–6 found in our case has not been described yet. Regarding the position of LMNA mutation, upstream mutations relative to the nuclear localization signal sequence, i.e., exons 1–6, were found to be significantly associated with an adverse cardiac phenotype [23,28].

In conclusion, we can presume that the high rate of arrythmias in our family may be due to the mutation localization and mechanism.

The first clinical manifestation in patients with *LMNA* mutations are often AV blocks and AF, rather than DCM or signs of heart failure [29,30] as could be observed in our case. Patient III:1 first presented with supraventricular tachycardia and developed AV block before deterioration of LV function and patient III:2 first exhibited AF. Initially, *LMNA* mutations were detected in patients with early-onset conduction disturbances and DCM [3,31]. Conduction disease and supraventricular arrhythmias are significantly more prevalent in mutation carriers than in other patients with DCM [1]. In approximately 33% of patients with DCM and AV block, an *LMNA* mutation can be found [31]. Disease progression can be observed in terms of progressive AV block [29] as in patient III:1. Moreover, one prospective study including 47 patients found only conduction disorders to be significantly associated with VTs [32]. As a result of simultaneously elevated arrhythmic risk, current guidelines recommend ICD rather than sole pacemaker implantation for patients with an indication for pacemaker therapy [33]. Furthermore, in patients with an LVEF <50% and AV block, CRT-D should be considered, according to the recently published guidelines on cardiac pacing 2021 [33].

It is known that LMNA mutation carriers are at high risk for SCD. In 299 affected patients, van Berlo et al. reported an SCD rate as high as 46% with a mean age of 46 years [4]. Critically, arrhythmic risk, including SCD, often precedes heart failure [4]. This is in line with our case, in which the mean age of SCD was 47.8 years. Rijsingen et al. identified four risk factors for ventricular arrhythmias: non-sustained VT, male sex, LVEF < 45% and non-missense mutation [5]. These were translated to the European Society for Cardiology Guidelines for the prevention of SCD 2015, which propose early consideration for primary prophylactic ICD implantation in *LMNA* mutation carriers and above-mentioned criteria [34]. Recently, a novel risk calculator was introduced by Wahbi et al. after analyzing 444 patients; this risk tool included not only the aforementioned criteria, but also AV block and the absolute LVEF [14]. Herewith, they developed a score to estimate the 5 year risk for SCD and proposed an optimal threshold of 7% to 10% to prompt ICD implantation. This score resulted in better discrimination of unnecessary ICD implantation vs. prevention of SCD as compared to the conventional ICD indication proposed in the guidelines of 2015 [14].

Carriers of LMNA mutations are at high risk of SCD and of death due to severe heart failure. As illustrated in this report, the initial presentation, there is conflicting evidence concerning the main reasons of death in patients with LMNA mutations. Penetrance is generally high and increases with age. DCM develops usually after the age of 20 [30]. For example, in another retrospective study evaluating a total of 269 patients, heart failure was shown to be the most important reason of death, whereas approximately one third of patients died of SCD [5]. Heart transplantation was shown to be over-represented when compared to other DCM patients [29].

The imaging features of *LMNA* cardiomyopathy are often not discernible from other forms of DCM. Left ventricular function of *LMNA* mutation carriers and DCM does not significantly differ from non-*LMNA* DCM, whereas LVEDD tends to be lower than in non-*LMNA* DCM [1]. However, direct comparison of echocardiographic parameters with non-*LMNA* DCM is scarce and *LMNA* cardiomyopathy may also adopt the arrhythmogenic right ventricular cardiomyopathy phenotype [35]. *LMNA* cardiomyopathy show typical midmyocardial septal late-gadolinium enhancement (LGE) on cardiac MRI [29,36,37,38] as has been seen in patients 2 and 3 in our study. LGE presence seems more pronounced in DCM with *LMNA* mutation than in non-*LMNA*-DCM [37]. Holmström et al. described the presence of LGE in 88% of 17 mutation carriers with DCM, whereas Marco et al. described the presence of LGE in 44% of cases in a meta-analysis of 1305 patients with non-*LMNA*-DCM [37,39]. Augusto et al. showed a similar distribution of scar tissue mainly in the midwall septum and at the inferior insertion point of the right ventricle in most *LMNA*-carriers with DCM; however, findings failed to discriminate different DCM genotypes [40]. It has been hypothesized that septum fibrosis may be an underlying mechanism for conduction disturbances [6]. Peretto et al. could demonstrate a significant association between the presence of LGE and malignant arrhythmic events in a cohort of 41 patients with an *LMNA* mutation [38]. Interestingly, no patient without LGE developed VT or ventricular fibrillation at a mean follow-up of 10 years [38]. However, the small study sample may hamper transferability, and further studies are required to elucidate the role of LGE and its possible value for current risk scores.

In conclusion, our study further emphasizes the need for family screening, close follow-up visits and early risk calculation for SCD in patients with an *LMNA* mutation. Most notably, early onset of conduction disturbances and supraventricular arrhythmias should raise awareness and initiation of genetic counseling.

## Supplementary Materials

The following is available online at https://www.mdpi.com/article/10.3390/genes13020169/s1, Video S1: Echocardiographic samples of patient III:3; A 4-chamber view; B: 2-chamber view; C: short axis view.
